# Pre-Existing Inequality: The Impact of COVID-19 on Medicare Home Health Beneficiaries

**DOI:** 10.1177/1084822321992380

**Published:** 2021-05

**Authors:** William Cabin

**Affiliations:** 1 Temple University, Philadelphia, PA, USA

**Keywords:** COVID-19, Medicare, home health, home health nurses

## Abstract

There is significant data on the adverse impact of COVID-19 on persons who were poor, minorities, had compromised physical or mental health, or other vulnerabilities prior to the COVID-19 pandemic. A significant portion of the overall Medicare population has such vulnerabilities. The Medicare home health beneficiary population is even more vulnerable than the overall Medicare population based on gender, race, income level, living alone status, and number of chronic conditions. A literature review indicates there is only 1 study on the impact of COVID-19 in Medicare home health on home care workers and none on the impact on home health beneficiaries. The current study is a qualitative study based on interviews of a convenience sample of 48 home care nurses from 9 different home health agencies in New York City between April 1 and August 31, 2020. Six major themes emerged: need for social service supports increased; loneliness and depression increased among patients; physical and mental health conditions became exacerbated; substance use and abuse increased; evidence of domestic violence against patients increased; and there was a limited amount of staff and equipment to care for patients.

## Introduction

The purpose of the study presented in this article is to address a gap in the existing literature on the impact of COVID-19 on Medicare home heath beneficiaries.

There is significant evidence that the COVID-19 pandemic has had adverse impacts on many individuals who experienced adverse impacts of inequality related to race, gender, age, or income prior to COVID-19. Long et al.^1^ found such adverse impacts on gender and racial minorities regarding job losses with Hispanics having the steepest initial job losses; African Americans have recovered just over a third of their jobs lost in the pandemic compared to White Americans recovering more than half of their jobs; women lost more jobs than men; and African American women faced the largest setback in job losses. Adhikari et al.^2^ found infection rate and death rate disparities by poverty level and race in a study of ten combined statistical areas, all of which were urban areas. In comparing high poverty level counties which were substantially White to substantially non-White, they found the non-White counties had an infection rate nearly 8 times higher and a death rate more than 9 times greater. Overall, they found that the higher the poverty level of a county, regardless of race distribution, the higher the infection and death rate.

Leopold^3^ found in a New York City study that COVID-19 death rates in poor neighborhoods were more than 2.5 times higher than in wealthier neighborhoods. Leopold^3^ also found that African-Americans and persons born in Latin America had 9.0 and 18.4 more deaths per 100,000 than Whites, respectively; low-income persons had 27.6 more deaths per 100,000 than higher income persons; and the elderly had 23.2 more deaths per 100,000 than persons of other ages. The Centers for Disease Control and Prevention^4^ has reported that the elderly had the highest rate ratios of any age category for both COVID-19 hospitalization and death compared to the 18- to 29-year old category. For hospitalizations, the 65- to 74-year old hospitalization rate was 5 times higher, for the 75- to 84-year old category the rate was 8 times higher, and for 85plus year old category the rate was13 times higher. For deaths, the 65- to 74-year old hospitalization rate was 90 times higher, for the 75- to 84-year old category the rate was 220 times higher, and for 85plus year old category the rate was 630 times higher.

The PAN Foundation, using federal data, found COVID-19 has had a disproportional impact on older adults.^5^ They found, as of April 30, 2020, 30.7% of all Covid-19 deaths in the United States were for persons 85 and older and that another 27.3% for persons 75 to 84 years old. They also found, based on a 27-state analysis, the Covid-19 death rate for African Americans was 2.7 times higher than Whites and that while African Americans represented 13% of the population in the 27 states, they represented 28% of the deaths in those states.

In a study of Medicare beneficiaries, Davis and Willink^6^ found that low-income Medicare beneficiaries had greater risks pre-COVID that were increased by financial hardships resulting from job and other financial losses related to COVID-19. They found that 28% of all Medicare beneficiaries are low-income, meaning their income is less than 150% of the Federal Poverty Level (FPL). Further emphasizing their limited financial resources, the study found 57% of the low-income beneficiaries also were dual eligible, meaning they had both Medicare and Medicaid, compared to only 19% of all Medicare beneficiaries. These low-income Medicare beneficiaries are at higher risk for short-term and longer-term COVID-19 consequences because of their characteristics. They found that 51% of low-income beneficiaries had 3 or more chronic conditions compared to 47% for all Medicare beneficiaries; 35% had percutaneous coronary intervention (PCI), otherwise known as angioplasty with stent, compared to 24% for all Medicare beneficiaries; and 7% had nursing home or other institutional care compared to 4% for all Medicare beneficiaries.

The Kaiser Family Foundation^7^ also found that a significant portion of Medicare beneficiaries had limited financial resources prior to COVID-19 and these resources have become more limited since COVID-19. More specifically, they found: half of all Medicare beneficiaries lived on incomes below $29,650 per person in 2019 and 1 in 4 had incomes below $17,000 per person; half of all Medicare beneficiaries had savings below $73,800 per person in 2019, one fourth had less than $8,500 per person in savings, and 12% had no savings or were in debt; and half of all Medicare beneficiaries had home equity below $73,350 per person in 2019, and one fourth (27%) had no home equity at all. In a survey, Kaiser also found “Nearly half of older Americans (48%) say they are worried that their investments will be negatively affected by coronavirus disease and the spillover effects of ongoing market volatility on their economic security.”

The Centers for Medicare and Medicaid Services,^8^ in a July 28, 2020 update, reinforced the disproportional impact of COVID-19 on racial and ethnic minorities, stating “The updated data confirm that the COVID-19 public health emergency is disproportionately affecting vulnerable populations, particularly racial and ethnic minorities. This is due, in part, to the higher rates of chronic health conditions in these populations and issues related to the social determinants of health.”^8^

## Literature Review

The literature review used Cinahl, PubMed, Medline, Cochrane Library, Campbell Collaboration, PsycINFO, Sociological Abstracts, and Social Science Abstracts databases with a search period of January 1, 2019 through March 31, 2020 followed by an updated search after the study was conducted covering April 1, 2020 through November 30, 2020. Multiple keywords were used in the search: COVID 19; COVID; COVID 19 and home care; COVID and home care; COVID and home health; COVID 19 and home health; COVID and Medicare; COVID-19 and Medicare. The search yielded multiple studies and data on Medicare and COVID-19. The search yielded multiple citations on Medicare home health, but only 1 issue brief and 2 studies.

Of the 2 studies, there was only 1 that used interviews of home health staff, patients, or caregivers. The 1 study interviewed 33 home care home health and personal care aides and home attendants in 24 different home health agencies in the 5 boroughs of New York City.^9^ The study found these workers: were on the front lines of the COVID 19 pandemic but felt invisible; reported a higher risk for virus transmission; received varying amounts of supplies, information, and training; relied on non-agency sources for support; and were forced to make difficult tradeoffs in work and their personal lives.

The second study^10^ analyzed 1409 Medicare home health patients admitted for services at the Visiting Nurse Service of New York (VNSNY) between April 1 and June 15, 2020. Based on VNSNY data found 94% of patients upon discharges had statistically significant improvements in symptoms and function. The issue brief^11^ used an unspecified number of interviews with unspecified persons at home health agencies for part of the brief but primarily discussed the structure of the Medicare home health benefit and COVID-related federal policy changes. The study found that COVID-19 infection rates among Medicare home health beneficiaries and the home health workforce “have not been systematically reported during the pandemic.” The study^10^ also found that federal policy changes “have provided financial support to home health agencies, expanded provider licensures to certify use of home health, facilitated wider use of telehealth, and increased flexibility in Medicare Advantage plans.”

## The Current Study

The condition of Medicare home health beneficiaries during the COVID-19 crisis was the focus of the current study because these beneficiaries are more vulnerable than Medicare beneficiaries in general and because of the lack of studies on their situation. Avalere Health^12^ found, based on 2017 Medicare data, that compared to all Medicare beneficiaries, Medicare home health beneficiaries are poorer (50.4% with income less than $25,000/year compared with 37.3% for all Medicare beneficiaries); and have more chronic conditions (47.3% with 5 or more chronic conditions compared with 22.4% of all Medicare beneficiaries with 5 or more chronic conditions). In addition, Avalere Health^12^ found that 27.8% of Medicare home health beneficiaries had 2 or more Activities of Daily Living limitations (ADLs) compared to 10% of all Medicare beneficiaries.

Food insecurity is another pre-existing condition for Medicare beneficiaries and even more for home health beneficiaries. A 2020 study using 2016 Medicare data found that 10% of Medicare beneficiaries were food insecure, with lower-income beneficiaries more likely to report food insecurity.^13^ While the study did not focus on home health beneficiaries the higher percentage of low-income beneficiaries in Medicare home health indicates they may have higher levels of food insecurity than Medicare beneficiaries in general.

Mental health issues have increased during COVID-19^14^ and mental health is a significant vulnerability for Medicare home health beneficiaries. CMS reported in 2017 that 38.3% of all Medicare home health beneficiaries had a severe mental illness (SMI), which is defined “as having depression or other mental disorder including bipolar disorder, schizophrenia, and other psychoses (p. 28).”^12^ This rate compared with only 28.3% among all Medicare beneficiaries (p. 28).^12^ Of those Medicare home health users with SMI, 96.2% had depression and 23.6% had an additional mental disorder. In addition 57% of Medicare home health beneficiaries had an income less than 200% of the Federal Poverty Level (FPL) compared to 44% of all Medicare beneficiaries and 27% had an income less than 100% of the FPL compared to 18% of all Medicare beneficiaries (p. 12).^12^

Home health nurses were selected as the lens for beneficiary experiences because they represent the most visits of any professional in Medicare home health, with 48 % of all national Medicare home health visits in 2017, and the highest number of visits per episode, with 8.2 in 2018 and 8.4 in 2017; conduct most initial home health patient assessment visits; and develop and oversee most Medicare home health plans of treatment.^15,16^

## Methods

The study used a grounded theory approach.^17^ Grounded theory is the research methodology of choice because it was developed for interpreting qualitative data in the absence of a pre-existing theory. In the present study, the existing literature does not provide insight into how home care nurses perceive the impact of COVID 19 on their homebound beneficiary population. Data were collected through interviews of 48 home care nurses from 9 different Medicare-certified home care agencies in the 5 boroughs of New York City between April 1, 2020 and August 31, 2020. All interviews were conducted virtually by computer, using Zoom software, and an interview guide was used to help standardize the data collection. Participants were selected using a snowball convenience sampling technique, whereby home care industry professionals known to the author identiﬁed potential interviewees. In-person interviews were conducted at locations convenient to participants and off-site from where they worked. An interview guide was used to help standardize the data collection. The study was self-funded by the researcher and therefore not subject to any IRB approval. However, all study participants received and signed informed consents written in compliance with federal regulations and all participants were assured of anonymity and conﬁdentiality. Qualitative analysis began shortly after the initial data were collected and resulted in additional questions and probes that were applied to subsequent interviews, in an ongoing iterative process. Analysis followed the grounded theory three-stage coding of interview data: open, axial, and selective coding.

Open coding was used to fracture the data to “identify some categories, their properties, and dimensional locations.”^17^ The coding and classiﬁcation generated a list of 238 codes. Code and category labels were created, systematically sorted, compared, and contrasted until they were complete, with no new codes or categories produced and all data accounted for. Through axial coding, multiple phenomena were identiﬁed from the connected categories and subcategories. These phenomena included the nature of the beneficiary population in terms of vulnerability factors such as income, living alone status, gender, race and number of chronic conditions; availability of information, supplies, and training to deal with COVID; social isolation, depression, and loneliness among beneficiaries; physical and mental health conditions among beneficiaries; and evidence of physical and emotional abuse among beneficiaries. Finally, using selective coding, a “story line” was identiﬁed and a “story” written that integrated the axial coding phenomena.^17^ The story that emerged was the adverse impact of COVID 19 on a Medicare home health population composed largely of persons with significant vulnerabilities due to the impacts of inequality pre-COVID 19.

In keeping with the grounded theory approach, the data analysis and interpretation were facilitated by analytical and self-reﬂective memo writing, which helped move empirical data to a conceptual level; expanded and reﬁned the data and codes; developed core categories and interrelationships; and integrated the experiences, interactions, and processes embodied in the data.^17^ All initial abstraction, analysis, and interpretation were done by the author of this article. After the initial process, all abstraction, analysis, and interpretations were reviewed by 2 additional experienced qualitative researchers, each of whom had a doctoral degree in social work and more than 15 years’ experience doing government-funded qualitative research on substance abuse. Any differences were discussed by the 2 external reviewers and the author to reach final decisions used for the study results. All analyses were done using ATLAS.ti software.

Limited demographic data was collected from study participants using a short survey. The results appear in Table 1. Overall, the nurses were 45 to 55 years old (81%); female (95%); Caucasian non-Hispanic (81%); had 6 to 10 years of home care experience (80%); and had an average caseload of 20 to 25 patients (83%). Statistical analysis of the demographic variables’ impact on study outcomes was not done due to the qualitative nature of the study.

**Table 1.** Nurse Participant Demographic Characteristics.

Characteristic	Number	Percent
Gender		
Male	2	5%
Female	46	95%
Race/ethnicity		
Caucasian, Non-Hispanic	39	81%
Hispanic	3	6%
African American	3	6%
Asian American	2	4%
Other	1	3%
Age range		
>55	2	5%
45-55	39	81%
36-44	4	8%
25-35	3	6%
Years as a home care nurse		
>10	4	8%
6-10	38	80%
1-5	5	9%
<1	1	3%
Average patient caseload		
26-30	4	8.5%
20-25	40	83%
<20	4	8.5%

## Results

Six major themes emerged: need for social service supports increased loneliness and depression increased among patients; physical and mental health conditions became exacerbated; substance use and abuse increased; evidence of domestic violence against patients increased; and there was a limited amount of staff and equipment to care for patients.

The themes were consistent across all nurses, regardless of their demographic characteristics (Table 1) or agency. The themes from interviews are detailed below with supporting quotes.

***Need for Social Service Supports Increased. “These are largely poor, isolated people who always were limited. Their needs pre-existed COVID. Now it is horribly worse.”***Nurse SH

That quote was from Nurse SH who continued saying:

I do not understand why anyone is surprised. Maybe it is because no policy people ever paid attention before. This is a poor, homebound population that is only allowed to receive limited acute care, which has been mainly nursing and physical, speech, and occupational therapy. Many of their needs are not addressed like transportation, food, and personal care assistance. These unmet needs existed before COVID. They just became more noticeable and worse because of COVID, but COVID did not cause them. Nurse SH

Other nurses agreed with Nurse SH that patient unmet needs for social supports pre-existed COVID.

Yes, that is true. Our patients are some of the most limited in Medicare. They are poor, have multiple chronic conditions, lots of ADL (Activities of Daily Living) limitations, and are homebound. They are much worse than your average Medicare recipient, except maybe some in nursing homes. I know. I have worked in hospitals, primary care, and nursing homes, and yet we do very little for them given their unique condition and situation. It always has been this way so, of course, a pandemic that poses high risks for the elderly will be even worse for home health patients. Nurse RD

Nurse TH emphasized the lack of social services pre-COVID:

Are you kidding me? These [Medicare home health] patients have been neglected by Medicare for years. They have significant social needs; they need a social worker or case manager to work with them on accessing food, transportation, housing assistance, getting groceries, much of which is available through government programs or non-profits, but it is not covered. Social workers can barely do anything; not even much therapy, let alone help in a meaningful way with accessing these services. Nurse TH

The lack of such social needs services to deal with the social determinants of health and lack of social work coverage has been documented in peer-reviewed journals in addition to the nurses’ perceptions.^18,19^ MedPac reported that Medicare home health social work visits historically represent the smallest percentage of all 6 Medicare home health services nationally and have been on a steady decline from 0.3 of all Medicare home health visits per episode in 1998 to 0.1 in 2018, for a 36% decline.^15^

***Social Isolation, Loneliness, and Depression Increased: “It is so normal for our patients to be isolated, lonely and depressed. Of course, it would be worse with COVID restrictions on social interaction.”***Nurse JN

Nurses also emphasized that social isolation, loneliness, and depression increased during COVID, but were always the norm, reinforcing the pre-existing conditions theme.

Every one of my patients, for years now, has been lonely, depressed, or isolated, or some combination of the three. How could you not. These are homebound elderly patients. We all learned in nursing school that these conditions are normal for the elderly. The only issue is the degree, and it definitely is worse if you are homebound. Nurse JN

For some of my patients my visit is their social life. And that is the same for many of their caregivers, most of whom are also elderly, even some also being on [Medicare] home health. Then add in COVID and limits on my visits and our [home health] aides and their social world and resources shrink to a tiny work. They become more isolated and, of course, depressed and worse. Nurse TW

Everyone complains about social distancing, social gathering restrictions, and the whole mask thing with COVID. It is all over the news; has been since February [2020] and continues to be so. Most of the people who discuss these issues are relatively healthy working, mobile, self-sufficient people. Can you imagine the impact on people, like our patients, who are not only medically compromised, but socially compromised? Nurse LR

***Physical and Mental Health Conditions Became Exacerbated: “This is serious stuff and always has been serious, but Medicare ignores our patients’ mental health needs, and many physical health needs. It is just worse now with COVID.”***Nurse KD

Nurse KD continued, noting the mental health concerns went well beyond depression, loneliness, and social isolation.

Medicare [home health] always ignores mental health. We barely assess for it in the OASIS [Outcome and Assessment Information Set] and, to the extent we do, it is limited and has only been in the last few years. Even if we detect metal health issues there is no requirement to treat them, it doesn’t affect our reimbursement, and we are not graded in the Home Care Compare on it. And even if we were, we have no resources. The social workers are so limited in what they can do [by the Medicare manual and regulations] that there is no mental health care. Yes, our patients’ mental health became worse during COVID, but it always became worse even before COVID. How could it not? We ignore it. Nurse KD

Oh my God, mental health. It is a no brainer. We [in Medicare home health] don’t do it. We do not assess it, the social workers are not allowed to provide it, and nurses are not trained in it. So, of course our patients’ mental health worsens. “It happened pre-COVID and even worse during COVID. It is bad.” Nurse TK

I agree mental health care does not happen. Just as bad is physical health. That is our focus—acute episodes for heart, respiratory, orthopedic, diabetes problems mainly. But we do not even do that well. Evidence? Well we have a lot of frequent flyers, high re-admission rates and high rates of discharge to hospitals or nursing homes. How can that be if we are doing a good job. I know some of it may be hospitals discharging too soon, but my experience is that our care is limited by financial concerns and coverage requirements thar limit the number, type and duration of our visits. So patients’ conditions exacerbate. It became worse with COVID because of staff shortages and lack of proper protections like masks and equipment. Nurse LH

***Substance Use and Abuse Increased: “We do not deal well with substance use or abuse and it became so much worse with COVID.”***Nurse TG

Nurse TG continued:

We do not assess or treat alcohol or other substance use. It may come up in an assessment or visit but we do not use any professional tool to assess it. Even if we detect it, we do not treat it. We [nurses] are not equipped to do that and we are focused on the primary diagnosis, the physical health acute need, not on substance use. It is frustrating because the two situations [physical health and substance use] are often related and failure to effectively treat one often exacerbates the other. Nurse TG

Other nurses emphasized the normal pre-COVID inter-relationship between mental health, physical health, and substance abuse and the worsening situation during COVID.

Yes, it always has been an issue. We do not treat or assess SUD [Substance Use Disorder]. It is not just this agency. I have worked in six home health agencies. It was the same. I have friends who have worked in multiple agencies for decades and it is the same. We are not required to assess for it, our reimbursement isn’t affected, most nurses are not trained to deal with it, and the social workers, who have the best training, are so restricted by regulations that they cannot help. So, there is no care. We can’t even help them [the patients] get to outpatient substance use therapy and they are homebound! Nurse SA

Alcohol use has become worse during COVID. We always had a lot of patients with alcoholism, but I think it became even more of a crutch, a help to deal with the pressures of COVID. I think it contributed to more frustration, anger and abuse between caregivers and patients even though I think they took it [the alcohol] to relieve the anger, frustration, depression and isolation. That is the irony of drug dependence isn’t it? Nurse KO

Other studies have fund Medicare home health lacking in its assessment and treatment of substance use and abuse pre-COVID.^20^

***Evidence of Domestic Violence Increased: “Domestic violence definitely increased during COVID. It was always there, but COVID created more isolation and stress. So you are homebound. Who do you take it out on?”*** Nurse KO

Nurse KO continued from her discussion regarding increased substance use and her belief it related to COVID.

It is always a challenge to identify domestic violence. Sometimes a patient or caregiver will mention it, but most do not. Sometimes you will see physical evidence, like bruises, but then you never know if it was from a slip or fall. It is a challenge, but I have seen more physical bruises and had more patients tell me they have been abused [during COVID] than before. Nurse KO

I definitely have had more patients tell me of [alleged] abuse by their spouse or a caregiver during COVID. It is so subjective, especially with o many of our patients having memory or cognitive issues. We are supposed to report it to the local elder abuse agency if we believe it but that is where the judgment and subjectivity comes in. Nurse NM

It is always heart-wrenching to hear these accounts, especially if you see some physical bruises, but I am a nurse with limited expertise in these issues. I am not trained to talk to patients in a professional way. I might re-traumatize them by using the wrong language. We have no psychiatric nurses to deal with this and the social workers are limited [by Medicare] in what they can do and we have fewer of them [the social workers] due to COVID. Nurse RS.

OASIS does not gather data on alleged domestic violence in Medicare home health and the Centers for Medicare and Medicaid Services (CMS) does not otherwise collect such data. There is limited research on the nature and extent of domestic violence witnessed, recorded or reported by home care workers.^21^

*There Was A Limited Amount of Staff and Equipment for Caring for Patients: “These patients are poor and are barely getting by. Most patients did not have any protective masks. We had to provide them or not visit.”* Nurse MS

Nurses emphasized the connection between patients pre-existing health, social, and economic situation to adverse impacts of COVID. As Nurse MS said:

Our patients have limited means. Most rely on social security for most of their income so they do not have a lot of spare change. We went in often with limited masks and protective gear, but it did not matter in some cases because the patients had no masks. The patients could not afford them or could not get out to get them. After all, they are homebound. Otherwise they would not be eligible. We had to either bring masks or not visit. Our supplies were limited so it often was a challenge. Nurse MS

Other nurses agreed the COVID situation “Just made thins worse. They already were bad,” according to Nurse FG, who continued saying:

We often were short staff and equipment. That made life worse for the patients. They already were in bad shape being poor, homebound, and physically and mentally very sick, much more than your average Medicare patient. We made fewer [nursing] visits and I know the same thing happened with our [home health] aides and other providers. We either lacked staff or protective equipment, or both, or the patients had no protective gear, not even masks. Nurse FG

Nurse TG furthered the point observing:

Yes, we did some telehealth visits and that was helpful, but for homebound patients it is diminished care. The contact is limited, we barely get to see any of their home to assess environmental risks, talking by Zoom limits insights on mental and physical conditions. It really is a limited alternative. It is one thing to use telehealth that allows you to monitor blood pressure and vital signs, assuming your agency has that capability. It is another to try to use telehealth to treat and care for patients. It does not work well.

## Limitations

The study was a qualitative, exploratory study. As such it does not address causality and has several limitations including: small sample size; lack of random sampling for sample selection; and lack of a randomized controlled trial experimental design to test specific interventions against a control group. The study also is limited to 1 geographic area and based on interviews only of home care nurses and home care nurses who were accessed through the researcher’s contacts with home care nurses. As a qualitative study there also was no quantitative analysis of results by key demographic characteristics such as age, gender, years of experience in home health.

## Discussion and Conclusions

As previously noted, the limited research on Medicare home health and COVID-19 has focused on staffing and equipment limitations, the increased use of telehealth, federal financial assistance, increased flexibility of Medicare Advantage plans, and 1 study of home health patient functional and symptom outcomes upon home care discharge. These studies do not address how Medicare home health care during COVID time period has been affected by the very nature of the patient population and underlying limits on treating patients due to eligibility, coverage and reimbursement issues in Medicare home health. The nurses interviewed indicated that the underlying patient situations and Medicare home health requirements pre-existing COVID affected patient care, with COVID making the situation worse.

At least 1 issue brief,^11^ noted the increased flexibility of Medicare Advantage plans covering non-medical benefits (ie, meal delivery, non-medical transportation, home modifications, other social supports) in discussing COVID. However, the brief only stated the regulatory but did not cite any study linking the increased flexibility to improved Medicare home health patient care. As noted by the nurses interviewed, the Medicare home health population has always had, pre-COVID, significant needs which might benefit from expanded non-medical or social support benefits, even more than Medicare Advantage plan beneficiaries. However, only a small portion of all Medicare Advantage beneficiaries (5.2%) are home health users^12^ so the expansion of non-medical coverage in Medicare Advantage plans will benefit few home health beneficiaries. In addition, Medicare home health beneficiaries are a more vulnerable population in terms of social and environmental factors compared to all Medicare beneficiaries, as discussed earlier in this article.^12^

The COVID-19 phenomenon clearly impacted Medicare home health as it did all of society. However, the narrow and limited focus on COVID-19 impacts on home health agency staffing, protective equipment and financing seems to avoid the underlying failure of the Medicare home health benefit to address the care needs of its beneficiaries. As the nurses observed, these unmet needs pre-existed COVID and, without policy changes, will continue during and after the COVID time period or, for that matter, any other medical or other crisis. Given the themes that emerged in this study, it seems policymakers should seriously consider revising the Medicare home health benefit to better address the unmet needs. Based on the nurses’ insights the initial revisions might best focus on expansion of the home health social work benefit; requiring substance abuse assessment and treatment; revising the OASIS to assess more mental health conditions; revising regulations to require treatment of assessed mental health and substance abuse needs; and allowing home health agencies the same flexibility in addressing social needs as they recently have granted Medicare Advantage plans.
